# Differential immune infiltrates in histomorphologic Wilms tumor regions identify prognostic macrophages

**DOI:** 10.1016/j.omton.2026.201162

**Published:** 2026-02-25

**Authors:** Lukas Watzke, Francesca Palmisani, Maud Plaschka, Florian Halbritter, Branka Radic-Sarikas, Katrin Rezkalla, Leo Kager, Heinrich Kovar, Renate Kain, Martin Metzelder, Helena Sorger, Gabriele Amann, Michael Bergmann

**Affiliations:** 1 Department of Pediatric and Adolescent Surgery, Medical University of Vienna, 1090 Vienna, Austria; 2 Department of Pathology, Medical University of Vienna, 1090 Vienna, Austria; 3 St. Anna Children’s Cancer Research Institute (CCRI), 1090 Vienna, Austria; 4 St. Anna Children’s Hospital, Department of Pediatrics and Medical University Vienna Children’s Cancer Research Institute, 1090 Vienna, Austria; 5 Department of General Surgery, Division of Visceral Surgery, Medical University of Vienna, 1090 Vienna, Austria; 6 Comprehensive Center for Pediatrics, Medical University of Vienna, 1090 Vienna, Austria

**Keywords:** MT: Special Issue - advancements in pediatric cancer therapy, nephroblastoma, metastasis, relapse, prognosis, risk group stratification, tumor microenvironment, SIOP, macrophages

## Abstract

Wilms tumor (WT) is characterized by a unique ternary histology, including blastemal, epithelial, and mesenchymal elements. Although overall survival is high, relapse and metastasis affect not only high-risk but also intermediate-risk (IR) patients. We here analyzed immune cell infiltrates in relation to histomorphological tumor regions of patients treated with neo-adjuvant chemotherapy and evaluated their role for disease prognosis. We included 46 chemotherapy-treated WTs resected between 2002 and 2020, which affected 27 females and 19 males at a median age of 35.58 months. Tumor samples were re-evaluated resulting in 13 mixed, 12 regressive, 8 blastemal, 7 mesenchymal, and 6 epithelial subtypes. HALO was used for automated quantification of the immunohistochemical stainings. Immune markers abundance was highly dependent on the histomorphological region. We observed significantly lower amounts of CD4, CD8, and CD206 positive immune cells in the blastemal region, as compared to the mesenchymal region. Moreover, abundance of CD206, CD86, and CD68 positive immune cells in the mesenchymal and blastemal regions showed a significant association with prognosis and timing of relapse. In conclusion, WT displays region-specific differences in immune cell infiltration. Evaluating CD206, CD86, and CD68 expression in mesenchymal and blastemal regions might be valuable for improving IR patient stratification.

## Graphical abstract

**Figure:**
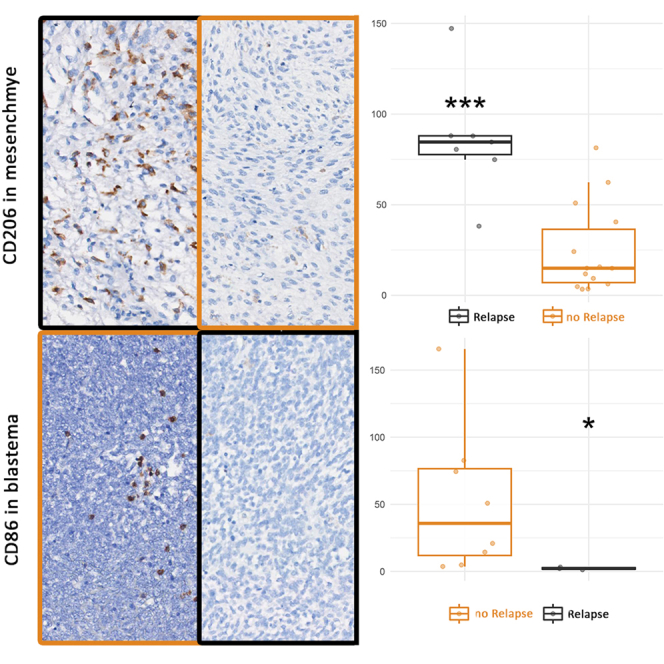

## Teaser

Wilms tumors show distinct immune microenvironments across histological regions. In 46 treated cases, HALO-based quantification revealed reduced immune infiltration in blastemal areas and prognostic relevance of CD206, CD86, and CD68 in blastemal and mesenchymal regions. Region-specific immune profiling can improve risk stratification beyond current histological classification.

## Introduction

Wilms tumor (WT) is the most common renal tumor in children and accounts for 6% of all childhood cancers.^1^^,^^2^ It arises from aberrations in fetal kidney development resulting in a unique histology, which can resemble various stages of the developing kidney entailing blastemal, epithelial, and mesenchymal components in heterogeneous compositions and varying degrees of differentiation.^3^ In this unique ternary pattern, blastema represents the most undifferentiated component with small blue round cell morphology and frequent mitotic figures. Epithelial cells tend to form higher organized patterns that vary from poorly differentiated rosette-like formations, to early and mature tubules and glomerulus-like structures. The mesenchymal component of WT is highly heterogeneous and known to develop into extreme states of cell differentiation, presenting as cartilage, bone, fat, muscle or hematopoietic cells.^4^ This tissue heterogeneity provides a unique possibility to study the spatial distribution and context-specific characteristics of immune cell infiltration within the tumor microenvironment (TME).

In order to treat WT, two different therapeutic strategies have evolved. In the European SIOP (Société International d’Oncologie Pédiatrique) treatment protocol, patients older than 6 months receive neo-adjuvant chemotherapy followed by surgical resection. They are subsequently categorized into low risk (LR), intermediate risk (IR) or high risk (HR) groups based on histology.^5^ The US-based COG (Children’s Oncology Group) guidelines, favor primary resection followed by adjuvant chemotherapy based on histopathological examination. Neo-adjuvant chemotherapy is restricted to a few selected cases.^6^ Both protocols, though different in approach, have improved survival rates to about 90% over the past decades.^7^^,^^8^

Despite a generally positive outcome, current patient stratification is imperfect, as only a third of relapsing or metastatic cases show HR features,^9^ leading to under-treatment in many cases. In these patients, 80% of relapses occur in the first 24 months after nephrectomy.^10^ In contrast, the extent of overtreatment remains unknown, but adverse late effects of intense chemotherapy in children have been well documented.^11^^,^^12^^,^^13^^,^^14^ Therefore, improving risk stratification is essential to avoid under- or over-treatment.

In many adult cancers, patient stratification has improved by studying the TME. The TME consists of non-malignant cells such as stromal and immune cells that influence therapy response and cancer progression.^15^ Targeting immune checkpoint proteins (ICPs) on T cells and immune cells has refined treatment and improved outcomes,^16^^,^^17^^,^^18^^,^^19^ with macrophages playing key regulatory roles: M1-like phenotypes support anti-tumor activity, whereas M2-like phenotypes promote stromal changes and correlate with poor prognosis.^20^^,^^21^^,^^22^^,^^23^^,^^24^ In contrast to adult cancers, WT and other pediatric solid tumors, have scarce immune cell infiltrates. While higher T cell counts show some prognostic benefits, trials with immune checkpoint inhibitors (ICIs) in pediatric cancers showed limited success.^25^ Most TME studies in WT focus on untreated (UT) samples,^26^^,^^27^^,^^28^^,^^29^^,^^30^ where higher T cell and M1-like macrophage counts are associated with better outcome, and higher M2-like macrophage counts are associated with poor prognosis.^28^^,^^29^^,^^30^^,^^31^ Wang et al. analyzed the role of tumor-associated macrophages (TAMs) for patient survival in both UT (*n* = 64) and pretreated (PT) (*n* = 84) WT and found a clear association of M2-like type macrophages with adverse prognosis in the UT cohort.^32^ Devendra et al. directly compared PT with UT and WT samples and found no difference in the distribution of tumor-infiltrating lymphocytes (TILs) among 17 PT and 8 UT samples.^27^ However, none of the aforementioned studies took into account the unique differences in histomorphological regions. With undifferentiated blastemal, partly differentiated epithelial components, as well as highly differentiated mesenchymal areas, WT provides a unique opportunity to study immune infiltrates across different stages of differentiation. Moreover, analysis of regressive or necrotic areas might contribute to our immunological understanding of treatment response.

We observed significant variations in the immune infiltrate across distinct histological regions of PT WT. The findings also had an impact on prognosis, highlighting tissue-based markers that could help refine current risk stratification methods.

## Results

### Rapid systematic review

To evaluate reported differences between PT and UT WT in relation to the immune TME, we conducted a systematic literature review, focusing particularly on studies evaluating tumor immune infiltrates and including clinical outcomes. It should be noted, that 50% of the selected studies focused exclusively on UT WT.^28^^,^^29^^,^^30^^,^^31^ Moreover, the majority of the studies focused only on a particular immune cell type, namely T cells or macrophages, beside one including general B cell, T cell, macrophage markers, and signaling proteins.^30^ One larger study analyzed the role of TAMs in patient survival in both UT (*n* = 64) and PT ( *n* =84) WT.^32^ They found decreased levels of M1 and M2 macrophages in clinical stages 3 and 4 PT WT samples. All other markers showed no significant differences in overall immune cell recruitment in UT compared to PT samples. Thus, they could not rule out and inhibitory effect of chemotherapy on macrophages. They also found a negative association of M2-type macrophages with survival in the UT and PT cohort. Additional three publications included both PT (combined number of patients, *n* = 21) and UT (combined number of patients, *n* = 162) WT.^26^^,^^27^^,^^33^ These studies did not find any significant differences in the pattern or amount of immune cell infiltration when comparing UT with PT samples. One of the studies found higher CD8 T cell densities to be associated with better outcome in both UT and PT WT; two publications found no prognostic difference for high vs. low CD68^+^ TAMs, while one publication did.^30^^,^^32^^,^^33^ One study directly compared PT with UT WT samples and found no difference in the distribution of TILs among 25 samples.^27^ Overall, our rapid review analysis demonstrates that UT and PT samples share a comparable immune landscape and pinpoints to macrophages as the predominant mesenchyme-located immune cell population of both UT and PT WT. It should be noted, that five publications found immune cells to be predominantly located in the tumor mesenchyme of WT.^28^^,^^29^^,^^30^^,^^31^^,^^33^ Four publications, analyzing only UT WT, found higher T cell and M1 macrophage counts to be positively associated with outcome, and higher M2 macrophage counts to be negatively associated with outcome.^28^^,^^29^^,^^30^^,^^31^

### Clinical data of the study population

Table 1 summarizes the clinical and histopathological characteristics for all 46 PT WT specimens that underwent immunohistochemistry (IHC) staining in this study. Age at diagnosis was highest among patients with mesenchymal tumors (49.81 months) and lowest among those with blastemal and epithelial dominant WT (26.4 and 27.5 months). One in a total of three patients with local relapse showed positive resection margins following partial nephrectomy, while the other two had tumor-free resection margins. Patients who developed distant metastases or relapsed were older at diagnosis than those who did not (44.35 months vs. 30.25 months, *p* < 0.001). Event-free survival did not differ between HR and IR patients. Among 46 patients, relapse occurred in 3 of 11 (27%) HR cases and in 10 of 35 (28%) IR cases. Fisher’s exact test indicated no significant difference in relapse frequency between the two groups (*p* = 1.00; odds ratio 1.06, 95% confidence interval [CI] 0.19–7.49). These findings suggest comparable event-free outcomes across risk categories in this cohort, although the wide CI reflects the small sample size and limited number of events. This pattern is consistent with the Kaplan-Meier analysis (Figure S1), which likewise demonstrated no detectable difference in event-free survival between the two groups.

**Table 1:** Clinical data

Characteristics	Histologic subtypes						Relapse or metastasis	
	all patients (*n* = 46)	blastemal (*n* = 8)	epithelial (*n* = 6)	mesenchymal (*n* = 7)	mixed (*n* = 13)	regressive (*n* = 12)	yes (*n* = 13)	no (*n* = 33)
Median age of patients at surgery (IQR^a^) – months	35.58 (30.7)	26.40 (14.71)	44.22 (41.43)	49.81 (23.03)	32.99 (22.21)	38.29 (21.15)	44.35 (23.75)	30.25 (31.44)
Female sex	27	4	4	4	10	5	6	21
Staging								
1	16	4	3	2	5	2	2	14
2	9	3	1	2	2	1	2	7
3	15	0	1	3	3	8	8	7
4	1	0	0	0	1	0	1	0
5	5	1	1	0	2	1	0	5
Risk group								
Low	0	0	0	0	0	0	0	0
Intermediate	35	2	5	7	9	12	10	25
High	11	6	1	0	4	0	3	8
Relapse and metastases								
Total events	13	2	0	2	3	6	13	0
Distant metastasis	12	1	0	2	3	6	12	0
Tumor relapse	3	1	0	1	1	0	3	0
Patients with pos. lymph nodes at nephrectomy	6	0	0	1	1	4	6	0
Scores								
Anaplasia	5	0	1	1	3	0	2	3
Mitosis								
None	14	1	1	1	2	9	4	9
Low (score of 1–2)	24	3	4	6	6	5	5	17
High (score of 3–4)	11	4	1	0	5	1	4	7
Deceased	4	0	0	1	2	1	4	0

a IQR denotes interquartile range.

### Different histomorphological WT subtypes display differential abundance of immune cell infiltrates

We first evaluated the spatial distribution of immune cell infiltrates within the distinct histomorphological regions of WT. To characterize the immune landscape in greater detail, we selected a panel of cell-specific markers: (1) CD4, CD8, and FoxP3 were used to identify various T cell subsets; (2) CD68 was used as a general marker of macrophages, CD86 and CD206 to distinguish between M1-like and M2-like polarized macrophages, respectively; and (3) CD20 was used to label B cells. In addition to these lineage-specific markers, we also included the functional ICP PD-L1 and IDO-1 (Indoleamine 2,3-dioxygenase), given their potential relevance for immunomodulatory therapeutic strategies. This comprehensive marker panel allowed us to assess both the composition and the potential functional state of the tumor-associated immune microenvironment.

We found distinct patterns of tumor-infiltrating immune cells (TICs) within the defined histomorphologic regions of WT. Blastema contained the lowest amount of TIC (median: 10 cells/mm^2^, interquartile range [IQR]: 28.9), while mesenchymal regions showed the highest amount of TIC per mm^2^ (median: 73 cells/mm^2^, IQR: 77.16), followed by epithelial regions of WT (median: 32 cells/mm^2^, IQR: 70.39). TIC density was significantly higher in mesenchymal compared to blastemal regions (*p* < 0.001), but not significantly different between mesenchymal and epithelial regions (*p* = 0.168) or between blastemal and epithelial regions (*p* = 0.305). Macrophages, as defined by CD68^+^ showed similar amounts of infiltrate when compared to lymphocytes (Wilcoxon rank-sum test: *p* = 1). CD20^+^ cells showed the lowest density in all tumor areas (median: 4 cells/mm^2^, IQR: 5.95), which was significantly lower than CD8^+^ or CD68^*+*^ cells *(p* = 0.001).

### T cells and B cells

**Figure 1:**
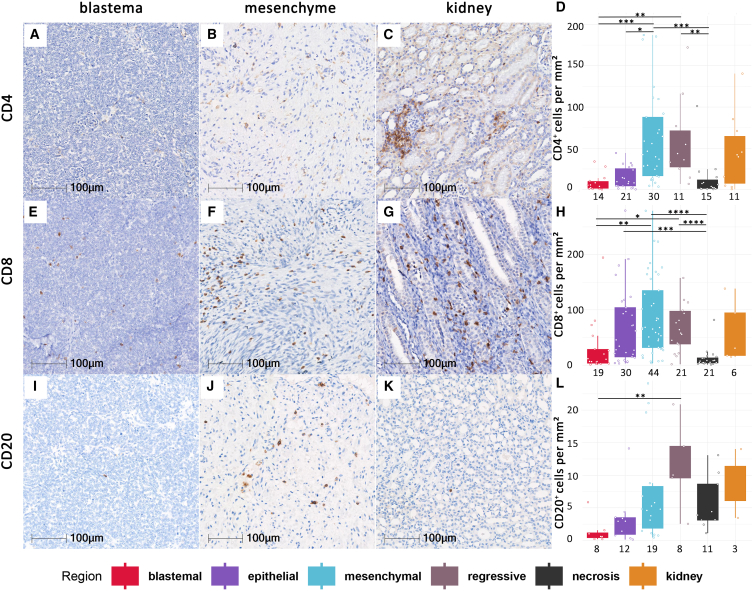
Expression of CD4, CD8, and CD20 in histomorphological WT regions Pictures display CD4 (A–C), CD8 (E–G), and CD20 (I–K). IHC-DAB-stained WT samples in blastemal (A, E, and I) as well as mesenchymal (B, F, and J) regions, and adjacent kidney (C, G, K) as internal controls. Graphs showing amount of CD4 (D), CD8 (H), and CD20. (L) IHC-DAB-stained cells in cells per mm^2^ in all analyzed subregions including blastemal, epithelial, mesenchymal, regressive, and necrotic areas as well as adjacent kidney tissue as internal controls. Patient numbers per region stained with CD4 (D) are 14 in blastemal, 16 in epithelial, 26 in mesenchymal, 10 in regressive, 15 in necrotic, and 9 in kidney tissue regions; for CD8 (H) 19 in blastemal, 28 in epithelial, 41 in mesenchymal, 18 in regressive, 21 in necrotic, 6 in kidney; and for CD20 (L) 8 in blastemal, 9 in epithelial, 16 in mesenchymal, 5 in regressive, 11 in necrotic, and 2 in kidney tissue. ∗*p* < 0.05, ∗∗*p* < 0.01, ∗∗∗*p* < 0.005, ∗∗∗∗*p* < 0.001.

With respect to T cells, mesenchymal regions (*p* < 0.001) and regressive regions (*p* = 0.001) contained significantly higher numbers of CD4 positive cells than blastemal regions. Mesenchymal regions contained significantly more CD4 positive cells than epithelial regions (*p* = 0.048). Similar results were observed for CD8 positive cells, where mesenchymal regions (*p* = 0.0023) and regressive regions (*p* = 0.032) contained significantly higher numbers than blastemal regions (Figure 1). There was no significant difference with respect to B cells, possibly because they were present only in low numbers.

### Macrophages

**Figure 2:**
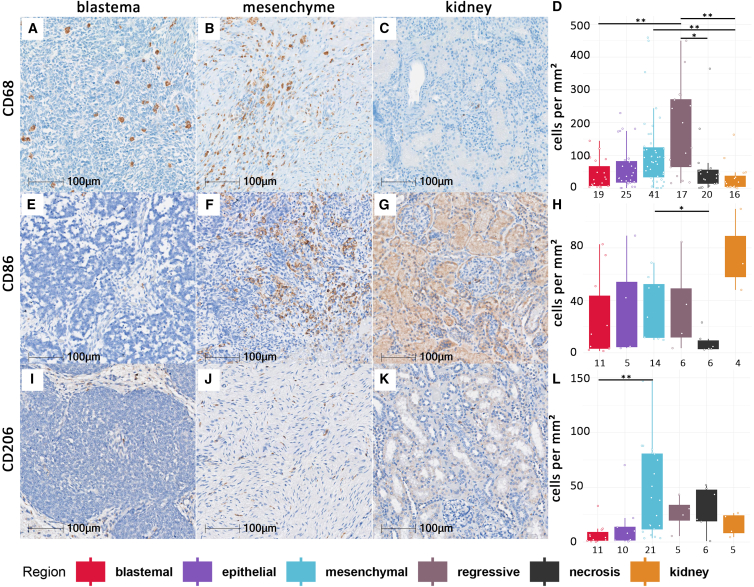
Expression of CD68, CD86, and CD206 in histomorphological WT regions Pictures display CD68 (A–C), CD86 (E–G), and CD206 (I–K) IHC-DAB-stained WT samples in blastemal (A, E, and I) as well as mesenchymal (B, F, and J) regions, and adjacent kidney (C, G, and K) as internal controls. Graphs showing amount of CD68 (D), CD86 (H), and CD206 (L) IHC-DAB-stained cells in cells per mm^2^ in all analyzed subregions including blastemal, epithelial, mesenchymal, regressive, and necrotic areas as well as adjacent kidney tissue as internal controls. Patient numbers per region stained with CD68 (D) are 19 in blastemal, 25 in epithelial 41 in mesenchymal, 17 in regressive, 20 in necrotic, and 16 in kidney tissue regions; for CD86 (H), 11 in blastemal, 5 in epithelial, 14 in mesenchymal, 6 in regressive, 6 in necrotic, 4 in kidney; and for CD206 (L), 11 in blastemal, 10 in epithelial, 21 in mesenchymal, 5 in regressive, 6 in necrotic, and 5 in kidney tissue. ∗*p* < 0.05, ∗∗*p* < 0.01.

Among all macrophage markers analyzed, CD68 exhibited the highest expression (median: 52 cells/mm^2^, IQR: 67.5), which was significantly greater than that of CD206 (median: 13 cells/mm^2^, IQR: 40.36)—the marker with the lowest expression (*p* < 0.0001). There was no significant difference when comparing these two markers with CD86 (median: 28 cells/mm^2^, IQR: 41.84). Mesenchymal areas contained significantly more CD68^+^ macrophages than adjacent morphologically normal kidney regions (*p* = 0.005) (Figures 2A–2D). M2-like polarized macrophages, defined by cell surface expression of CD206 were most abundant in mesenchymal and lowest in blastemal tumor areas (*p =* 0.007) (Figures 2I–2P), whereas M1-like macrophages, defined by expression of CD86, were most common in mesenchymal areas when compared to necrotic areas, which exhibited the lowest amounts of CD86 positive cells (*p* = 0.039) (Figures 2E–2H).

### WT therapeutic response is associated with an immune infiltrate

By analyzing PT tumor specimens, we were able to characterize the immune infiltrate within regions that subsequently responded to therapy, either through regression or necrosis. Among all annotated regions, regressive areas exhibited the highest density of TICs, with a median of 95 cells/mm^2^ (IQR: 136.32). Within these areas, CD68^+^ cells were the most prevalent immune cell subtype and were significantly more abundant than CD20^+^ cells, which showed the lowest infiltration (*p* = 0.044). Interestingly, necrotic regions still displayed a considerable number of TICs (17 cells/mm^2^, IQR: 26), which was slightly more than blastemal areas (median: 10 cells/mm^2^, IQR: 28.9). In necrotic areas, CD68^+^ cells represented the predominant TIC phenotype, with significantly higher densities compared to CD4^+^ (*p* = 0.038) and CD8^+^ (*p* = 0.034) T cells. Histologically, CD4^+^ and CD8^+^ cells were primarily localized along the peripheral margins of necrotic regions, whereas CD68^+^ cells were sparsely distributed within the necrotic tissue itself. Although no statistically significant difference was observed between M1-like and M2-like macrophage infiltration in these areas, a notable numerical trend was evident, with median densities of 4 cells/mm^2^ (IQR: 6.13) for CD86^+^ (M1-like), 32 cells/mm^2^ (IQR: 28.3) for CD206^+^ (M2-like), and 42 cells/mm^2^ (IQR: 40.73) for total CD68^+^ macrophages. The distribution differences between M1-like and M2-like polarized macrophages align with their established roles: M1-like as an active phenotype in the tumor mesenchyme and M2-like as a reactive phenotype in regressive tumor areas.^34^ Together these findings imply the importance of broad immune cell infiltration for successful tumor defense and underscore the central role of macrophages as key players in effective tumor responses.

**Figure 3:**
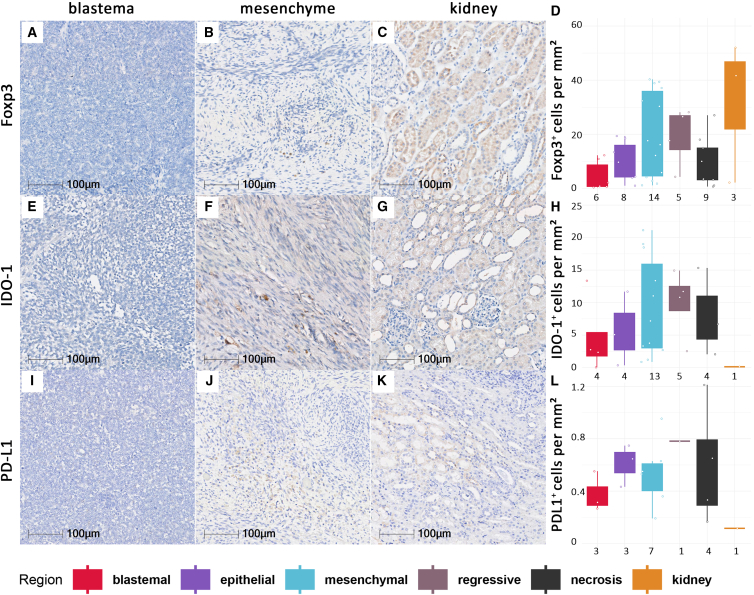
Expression of Foxp3, PD-L1, IDO-1 in histomorphological WT regions Pictures display Foxp3 (A–C), IDO-1 (E–G), and PD-L1 (I–K) IHC-DAB-stained WT samples in blastemal (A, E, and I) as well as mesenchymal (B, F, and J) regions and adjacent kidney (C, G, and K) as internal controls. Graphs showing amount of Foxp3 (D), DO-1 (H), and PD-L1 (L) IHC-DAB stained cells in cells per mm^2^ in all analyzed subregions including blastemal, epithelial, mesenchymal, regressive, and necrotic areas as well as adjacent kidney tissue as internal controls. Patient numbers per region stained with Foxp3 (D) are 6 in blastemal, 8 in epithelial 14 in mesenchymal, 5 in regressive, 9 in necrotic, and 3 in kidney tissue regions; for IDO-1 (H), 4 in blastemal, 4 in epithelial, 13 in mesenchymal, 5 in regressive, 4 in necrotic, and 1 in kidney; and for PD-L1 (L), 3 in blastemal, 3 in epithelial, 7 in mesenchymal, 1 in regressive, 4 in necrotic, and 1 in kidney tissue.

To classify infiltrating T cell subsets, we additionally stained for Foxp3, as a marker for regulatory T cells, as well as ICP, IDO-1, and PD-L1. Throughout all histomorphologic regions, T cells expressing Foxp3, IDO-1 or PD-L1 were rare (Figure 3). While the expression of Foxp3 and IDO-1 seemed to increase from blastemal to epithelial and mesenchymal regions (Figures 3A–3H), expression of PD-L1 remained under 1 positive cell per mm^2^ throughout all regions (Figures 3I–3L and S2).

### Macrophages are associated with metastatic disease in a time-dependent manner

**Figure 4:**
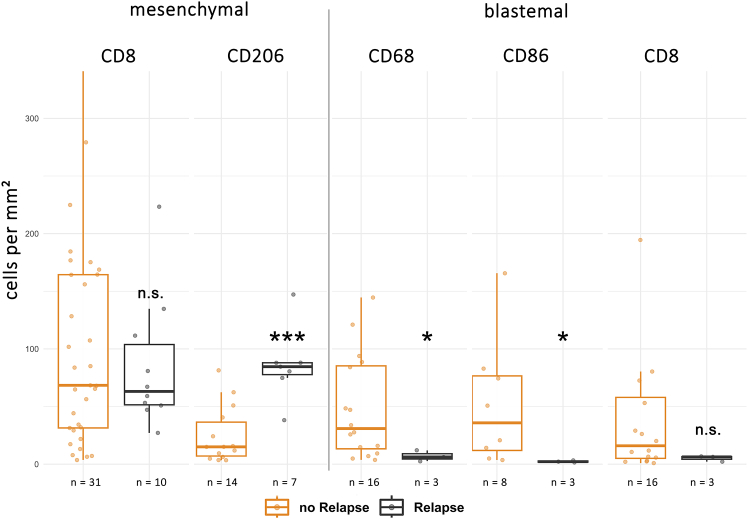
Macrophage and T cell counts in WT patients with and without relapse Boxplot graph showing amounts of CD8, CD68, CD86, and CD206 IHC-DAB-stained cells in mesenchymal and blastemal regions, comparing patients with relapse or metastatic disease and patients without relapse or metastatic disease. ∗*p* < 0.05, ∗∗*p* < 0.01, ∗∗∗*p* < 0.005, n.s., non-significant.

To evaluate the role of immune cells in patient prognosis, we compared immune infiltrates in WT of patients who developed distant metastasis or relapse (*n* = 13), to those of patients who remained free of disease for at least 4 years after nephrectomy (*n* = 33). Significant results in regard to prognosis were observed for immune cell counts in blastemal and mesenchymal regions. Patients who developed metastases or relapsed showed significantly lower counts of macrophages and M1-like polarized macrophages in blastema (CD68 *p* = 0.047, Cohen’s *d* = 0.98, 95% CI [−0.38, 2.34]; CD86 *p* = 0.012, Cohen’s *d* = 1.02, 95% CI [−0.58, 2.63]) and significantly higher counts of M2-like macrophages in mesenchymal tumor areas (CD206, *p* < 0.001, Cohen’s *d* = −2.25, 95% CI [−3.46, −1.04]) Figure 4). There was no significant, prognostically relevant difference observed for T cell infiltrates (blastema *p* = 0.254; mesenchyme *p* = 0.899), highlighting again the role of macrophages in disease progression.

**Figure 5:**
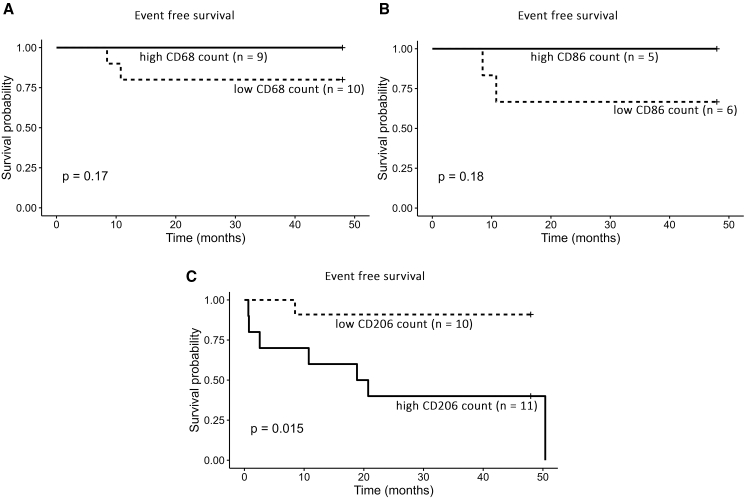
Event-free survival in patients with high- and low-macrophage counts Kaplan-Meier curves of event-free survival stratified by high vs. low immune cell infiltration for CD68 (A), CD86 (B), and CD206 (C). Cutoff values between “high” and “low” were defined by the median number of cells per mm^2^ (CD68: 26 cells/mm^2^; CD86: 14 cells/mm^2^; and CD206: 38 cells/mm^2^). While no statistically significant differences were observed for CD68 (*p* = 0.17) or CD86 (*p* = 0.18), high CD206 expression was significantly associated with reduced event-free survival (*p* = 0.015).

Analysis of event-free survival revealed that only mesenchymal CD206^+^ macrophages were significantly associated with relapse in a time-dependent manner. Over 50% of patients with high counts of mesenchymal resident M2-like macrophages, defined by cell counts greater than the median (CD206 = 38 cells/mm^2^), experienced relapse or distant metastasis within 2 years after nephrectomy (*p* = 0.015) (Figure 5). Blastemal CD68^+^ (*p* = 0.17) and CD86^+^ (*p* = 0.18) macrophages exhibited similar trends without reaching statistical significance (median cutoff values: CD68 = 26.03 cells/mm^2^, CD86 = 14.23 cells/mm^2^). Overall, macrophages emerged as the predominant immune cell population, whose presence correlated with relapse, with mesenchymal CD206^+^ macrophages in particular also correlating with the timing of relapse.

## Discussion

To our knowledge, this is the first study to characterize immune cell infiltrates across distinct histomorphological regions of WT, revealing a region-specific distribution of T cells, B cells, and macrophages. Overall, immune cell infiltration in vital tumor tissue was lowest in blastemal regions, followed by epithelial areas, and highest in mesenchymal tumor regions. These findings indicate a trend toward increased immune cell infiltration with higher levels of differentiation, ranging from the poorly differentiated blastema to the more differentiated epithelial and mesenchymal regions. A similar trend has been described in a mouse model for Ras/Myc-driven tumorigenesis, which was associated with T cell evasion and heightened expression of the CD206 M2-like macrophage phenotype.^35^

Given the limited sample size, the absence of a survival difference between IR and HR groups most likely reflects insufficient statistical power and should be interpreted with caution. At the same time, the continued pursuit of novel biomarkers remains fully warranted. Previous discoveries have demonstrated that advances in biomarker identification can substantially enhance patient stratification and guide therapeutic decision-making in WT.^36^ This need is further underscored by the observation that only approximately one-third of HR patients display histologic HR features, highlighting the importance of developing additional and more reliable biomarkers.^9^

Our study was solely conducted on chemotherapy PT samples. Previous studies comparing PT and UT WTs have consistently shown only minimal differences in the immune TME.^28^^,^^29^^,^^30^^,^^31^^,^^32^^,^^33^^,^^34^ Most analyses found comparable overall immune cell composition and localization, with macrophages representing the predominant mesenchymal immune population in both settings. While one report could not rule out a partial reduction in macrophage subsets after chemotherapy, the overall immune landscape appears largely preserved between UT and PT samples.^32^ In conclusion, the current literature supports that our findings in PT WT most likely reflect intrinsic features of the tumor rather than treatment-induced changes. Certainly, we cannot rule out a chemotherapy-related effect on the immune infiltrate within specific histomorphological regions, as a direct comparison with UT controls was not performed. However, looking at PT samples gave us an opportunity to determine the immune infiltrate on regressive regions, which usually are not observed in UT specimen, and hence most likely are the result of chemotherapy. Here, we found a predominance of macrophages in an M2-weighted distribution that fits our current understanding of macrophage polarization, where M1-like cells represent an active phenotype and M2-like cells as a reactive phenotype within regressive tumor regions.^34^ To our surprise, lymphocyte counts were still considerably high in regressive and necrotic regions, highlighting a functional potential within these compartments that has not yet been fully understood.

In our samples, CD4^+^ and CD8^+^ T cells exhibited highest density in mesenchymal and regressive areas and were most scarce in blastemal areas. In contrast to macrophages, which histologically appeared more evenly dispersed throughout the tumor tissue, CD4^+^ and CD8^+^ T cells showed local high-density clusters, accumulating mostly at the borders between regressive/necrotic regions and areas of viable tumor. While this could be interpreted as a pattern indicative of anti-tumoral activity, unlike other studies analyzing UT specimens, we did not observe any beneficial effects of higher T cell infiltrates on patient outcome in PT WT.^26^^,^^31^ These findings may reflect a shift toward a greater role of innate immunity in the post-chemotherapy TME of primary WT. The PD-L1 and IDO-1 ICP showed little to no expression, and unlike in adult cancers, do not seem to be relevant for functional suppression of these T cell clusters in WT. The origin and relevance of the observed T cell clusters in PT WT is yet to be determined and could be a subject for future studies.

In our study, the most abundant macrophage subtype in PT WT was CD68^+^. While Wang et al. found a co-expression of CD68 and CD163 beneficial for survival, we found that the CD206^+^ M2-like phenotype is correlated with relapse in a time-dependent manner (Figure 5).^32^

A lack of M1-like macrophages was associated with relapse and metastasis in our study. M1-like macrophages are activated through toll-like receptors and interferon-γ.^37^^,^^38^ They are potent killers of intracellular micro-organisms, secrete cytokines, and play a central role in on-site T cell activation through co-stimulatory surface proteins CD80 and CD86.^39^^,^^40^ Without these signals, T cells become inactive and are forced into apoptosis. M1-like macrophages also recruit T cells to the TME through the secretion of CXCL9 and CXCL10.^34^ In the context of WT, recruitment of CD86^+^ macrophages was shown to be a positive prognostic factor for UT WT, but not for PT WT patients.^32^ We show that blastema resident CD86^+^ macrophages are associated with better outcome in this subgroup of WT patients. Overall, levels of M1-like macrophages in the TME of WT were low in our study, which may in part explain the low T cell counts that WT and other pediatric solid tumors are known for.

Current clinical trials investigating new therapeutic options in WT mainly focus on the T cell component of the TME.^25^ Due to the inherently heterogeneous composition of WT and specifically its lack of T cells, a focus on macrophages and their phenotypes could be an appealing alternative. For example, therapeutically turning M2-like macrophages into M1-like macrophages has already shown promising results in osteosarcoma.^41^ Our results may imply that there is potential for a similar approach in the treatment of patients with PT WT.

The density of M2-like macrophages has been shown to increase with tumor stage and progression in UT tumors.^28^^,^^32^ We report here that the M2-like marker CD206 in mesenchymal regions of WT is strongly associated with poor prognosis in PT WT. These findings corroborate results from previous studies conducted in UT WT.^28^^,^^29^^,^^32^ Despite the small cohort size, which reflects the rarity of the disease, the difference between groups was marked, with an effect size greater than two standard deviations (Cohen’s *d* = −2.25). Such a large effect size supports the biological relevance of the finding and suggests that the same relationship would be expected to reach statistical significance in studies with greater sample size.

We therefore postulate that monitoring macrophage infiltrates in the tumor mesenchyme, as well as blastema, could contribute to a more accurate risk stratification of patients with IR WT in the future. Ideally, a combination of threshold values for CD206+ and CD86+ macrophages in the respective regions should be established and analyzed routinely. As demonstrated here, this could be achieved with the help of currently available powerful algorithmic digital pathology tools. A persistent challenge for risk stratification is posed by monophasic and especially epithelial WT, in which we did not detect any immune cell populations associated with prognosis in our study. However, epithelial-dominant WTs are characterized by specific genetic alterations, exhibit a favorable response to therapy, and are therefore classified as having favorable histology.^42^ In our study, the strongest association with prognosis also in a time-dependent manner was observed in mesenchymal areas. Mesenchymal tumor regions are present in the majority of PT WT, independent of histopathologic subtype and are therefore amendable to immune cell-based risk stratification in most patients with WT.

In summary, immune cell infiltrates in the histomorphological regions of WT follow a distinct pattern that is not only associated with the level of differentiation, but also could be utilized to optimize patient stratification. By quantifying immune cell infiltrates in histological subregions of WT, we showed that metastasis and relapse—the leading causes of death in these patients—are significantly associated with elevated CD206 expression in mesenchymal and reduced CD68 and CD86 expression in blastemal regions. Evaluating macrophage infiltrates in these WT regions could be used as a new add-on for future risk stratification assessment guidelines. Our study did not include UT WT samples and was based on a small sample size, which poses statistical limitations to our results and their interpretation. In the future, large multicenter studies could help confirm the importance of macrophage phenotyping in WT IR group stratification.

## Materials and methods

### Rapid systemic review

A rapid systematic review was conducted in 2024 in accordance with the Preferred Reporting Items for Systematic Review and Meta-Analysis (PRISMA) checklist to provide context for our data^43^ (Table S1). The Pubmed database was searched for mesh terms such as “Wilms tumor,” “tumor microenvironment,” “macrophages,” and “lymphocytes.” Applied filters included “humans,” “English,” and years “2000–2024.” In the case that a decision for inclusion could not be made based on abstracts alone, full-text articles were consulted. Articles that did not investigate the spatial distribution of immune cells were excluded. The total time invested for data acquisition according to the PRISMA workflow was 4 months, which was the quality limiting factor of this rapid systemic review.

### Cases/patients

This study was conducted following the principles stated in the Declaration of Helsinki and was approved by the ethics committee of the Medical University of Vienna (Ethics Commission vote no. 1640/2020). All patients included in this study were treated in accordance with the SIOP guidelines. H&E slides of 46 patients, who underwent nephrectomy between 2002 and 2020, were retrieved from the Pathology Department’s archive at the Medical University of Vienna (Table 1). Cases were reviewed by an experienced pediatric pathologist (G.A.), blinded to the patients’ diagnoses, and 1–2 representative whole slides per patient were selected for further investigations. During revision, one case of WT was re-classified as clear cell sarcoma of the kidney, and histological subtype diagnoses of 8 WT cases were re-categorized (4 mesenchymal as 3 regressive and 1 epithelial; 2 mixed as regressive; 1 regressive as mixed, and 1 blastemal as epithelial). One patient was downgraded retrospectively from high to IR. The final cohort consisted of 46 PT patients with 8 blastemal, 6 epithelial, 7 mesenchymal, 13 mixed, and 12 regressive WTs. A total of 12 patients developed distant metastases and 3 experienced local tumor relapses. Overall, 35 patients were histologically classified as IR and 11 as HR. All four patients who succumbed to their illness suffered either from metastasized disease or local tumor relapse after complete resection.

### Definition of histologic regions

**Figure 6:**
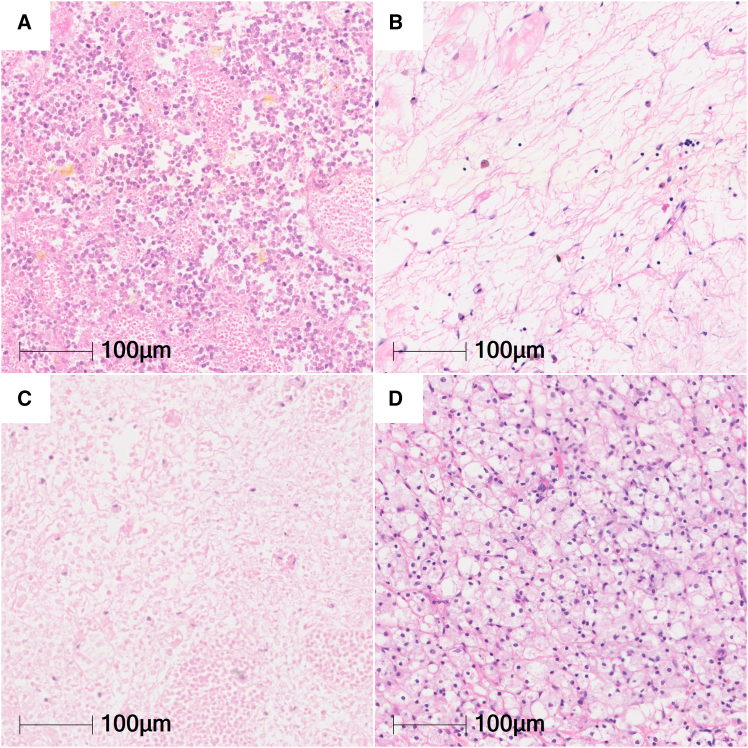
Necrotic and regressive WT areas Hematoxylin and eosin stain of WT samples post chemotherapy, devoid of nuclear hematoxylin stain, cytoplasmic membranes, and basic cellular structures in necrotic areas (A and C) and regressive changes with pronounced myxoid areas (B) and foam cell aggregates (D).

We defined blastemal, epithelial, and mesenchymal regions applying histopathological criteria outlined by SIOP guidelines.^44^ Regressive areas were defined as tumor areas including areas with foam cells and fibrosis most likely caused by neo-adjuvant therapy (Figure 6). To acknowledge that tumor regression is a dynamic process distinct from complete necrosis, which represents a final steady state, we analyzed necrotic and regressive areas separately.

### IHC

For IHC, formalin-fixed paraffin-embedded (FFPE) tissue blocks were cut into 4-μm-thick sections. A summary of the primary antibodies used and number of slides stained can be found in Table S2. R.T.U. Vectastain Elite ABC-HRP Kit (Vector Labs, Catalog no. PK-7200) was used as a secondary antibody for manual and Ultraview Universal 3, 3′-diaminobenzidine (DAB, Ventana) for automated staining protocols. To optimize IHC staining and dilutions of primary antibodies, all positive controls were established on tonsil tissue with the exception of IDO-1, which was tested on placental tissue. Negative controls were created by omitting the primary antibody during staining. In cases, where available, adjacent normal kidney was stained to provide an internal control of PT histomorphologically inconspicuous tissue. The median distance from tumor tissue to adjacent normal kidney tissue was 2 mm (2011.9 μm).

### Digital analysis

**Figure 7:**
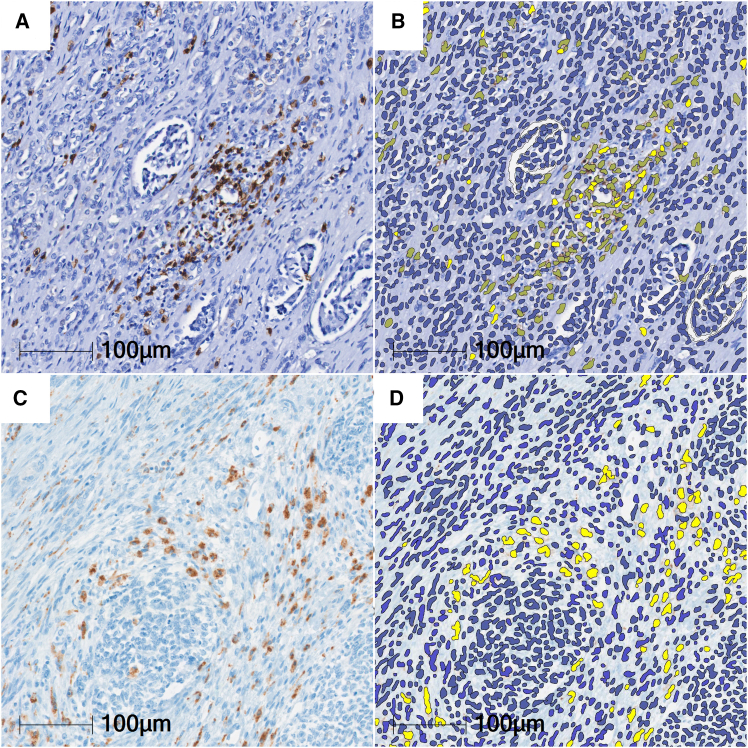
Multiplex IHC v.3.1.4 cell segmentation and phenotyping WT sample with CD8 (A and B) and CD68 staining (C and D) by immunohistochemistry using DAB as substrate. Overlay using HALO multiplex annotations (B and D) (bright and dark yellow: DAB-stained cells, blue: hematoxylin counter stain).

Stained slides were scanned in a Vectra Polaris whole slide scanner (PerkinElmer) using bright field scanning to provide digital images for further analysis. Digital analysis was carried out using the HALO AI v.3.1.4 software (Indica Labs). Histomorphologically distinct subregions of WT were manually annotated and defined as blastemal, epithelial, mesenchymal, regressive, necrotic, or adjacent kidney. Multiplex IHC v3.1.4 algorithm and Nuclei Phenotyper AI (Indica Labs) were trained to identify DAB positive cells and hematoxylin positive nuclei or areas of cells, representing immune cells and tumor cells (Figure 7). The algorithm was also trained to exclude immunohistochemical artifacts. DensenetV2 (Indica Labs) was trained to classify WT subregions of mesenchyme, blastema, and epithelium (Figures S3 and S4). In addition to these analyses, manual annotation was used to include regressive and necrotic areas, as well as adjacent non-neoplastic kidney tissue while excluding other artifacts such as air inclusions, folded or torn tissue, and dye precipitates. Training regions were expanded until AI annotation matched the primary observer’s (L.W.) annotation, which was confirmed by an experienced pediatric pathologist (G.A.). AI performance was then cross-checked by manual counting of randomly selected areas.

### Statistical analysis

All data were tested for normal distribution using histogram plotting as well as the Shapiro-Wilk test. Wilcoxon rank sum exact test was used to compare immune cell infiltrates between relapsed and non-relapsed patients. For multiple univariate testing, if not otherwise stated in the text, we conducted a one-sided Dunn’s test and subsequently applied Bonferroni correction to adjust *p* values. For independence testing of categorical variables, Fisher’s exact test was used. All statistical analysis was conducted in R project for statistical computing v.4.2.0 with a standard significance level of 0.05.

## Data availability

The datasets generated during the current study are available from the corresponding author on reasonable request.

## Acknowledgments

We are grateful to Margit Schmeidl, Barbara Neudert, and Michaela Schlederer for their assistance with archival work and immunohistochemistry. This project was funded by the Austrian Federal Ministry of Women, Science and Research and the Ludwig Boltzmann Gesellschaft (LBG) as part of the Clinical Research Group (CRG) programm (No. LBG_KFG_2024_105).

## Author contributions

Conceptualization, F.P., M.B., and G.A.; writing – original draft, L.W. and H.S.; investigation and methodology, L.W., F.P., and M.P.; supervision, G.A., M.B., M.M., H.K., B.R.-S., F.H., and H.S.; and writing – review and editing, M.B., R.K., H.K., F.H., and H.S.

## Declaration of interests

M.B. is an associate editor at *Molecular Therapy Oncology*.

## Supplemental information

Document S1. Figures S1–S4 and Tables S1–S2

Document S2. Article plus supplemental information
