# Supplementary material for: Differential immune infiltrates in histomorphologic Wilms tumor regions identify prognostic macrophages
Source: Mol Ther Oncol. 2026 Feb 25;34(1):201162. doi: 10.1016/j.omton.2026.201162 (PMC12997331; doi:10.1016/j.omton.2026.201162)
Supplement: Document S1. Figures S1–S4 and Tables S1–S2 [file mmc1.pdf]

OMTON, Volume *34*

Supplemental information

Differential immune infiltrates in histomorphologic Wilms tumor regions identify prognostic macrophages

Lukas Watzke, Francesca Palmisani, Maud Plaschka, Florian Halbritter, Branka Radic-Sarikas, Katrin Rezkalla, Leo Kager, Heinrich Kovar, Renate Kain, Martin Metzelder, Helena Sorger, Gabriele Amann, and Michael Bergmann

Table S1 - Rapid Review

FC-flow cytometry, HPF-high power field, IF- immunofluorescence, IHC-immunohistochemistry, MMP9-matrix metalloproteinase 9, TIL- tumor infiltrating lymphocytes, TMA- tissue micro-array, TME- tumor microenvironment, WB- western blot, RT-qPCR – real time q polymerase chain reaction

Reference no.	Year	Material	Method	Analysis	Patient no.	Study type	Markers	Findings
(1)	2020	FFPE, 5µm	IHC	no regions, necrosis excluded, manual counting 10 HPFs of intratumoral and invasive border	42 UT	retrospective	CD8	Better outcome with higher TIL score in center and border.
(2)	2019	Fresh tumor, FFPE, blood	FC, IHC	IHC counting methodology not disclosed	2 PT, 2 UT + 1 Denis Drash (UT)	Retrospective	CD4, CD8, PD-L1	No difference between infiltrates in PT and UT. CD8 positively correlates with outcome, weak PD-L1 in only 1 patient, CD4 and CD8 mostly in tumor mesenchyme.
(3)	2022	Bulk RNA, FFPE	IHC, WB	manual counting	72 UT	Retrospective, cell line	MMP9	MMP9 higher in M2, MMP9 predict poor survival.
(4)	2020	FFPE	WB, IHC, IF	3 HPF per sample, randomly selected	61 UT	Retrospective	CD80, CD163	M1 and M2 higher in tumor than adjacent, M1 and M2 mostly in stroma, M1 declined with higher stage, M2 increase with higher stage, no differences in sex, age, tumor location.
(5)	2014	FFPE, 5µm	IHC, IF	kidney-tumor, regions described without quantification, 10-15 representative HPF quantified by NIS-Elements Software	16 UT	Retrospective	CD3, CD20 CD68, COX-2, MPO, HIF-1a, ERK1/2, STAT3, iNOS, NT, VEGF	immune cells in stroma predominant, CD3 50 times more than in normal kidney, CD20 only in 7 patients, more CD68 than other immune cells.
(6)	2013	FFPE	IHC on 1-2 TMA cores per patient	digital analysis in adobe PS CS3	2 PT<br>152 UT	Retrospective	CD68	No difference in infiltrates between PT and UT. No overall survival difference in high vs low CD68 (only in stage 2), increased macrophages associated with microvascular invasion.
(7)	2019	FFPE, 3-5µm	IHC	center vs peritumoral in 10 randomly selected visual fields	17 PT<br>8 UT	Retrospective	CD3, CD4, CD8	No difference between infiltrates in PT and UT.
(8)	2024	FFPE	RT-qPCR, WB, IF	3 randomly selected 0,18mm² images analyzed per patient with ImageJ	64 PT<br>84 UT	Retrospective	CD68, CD163, CD80, CD86, CD206, Arg-1	No difference between overall infiltrates in PT and UT, but decreased M1/M2 in stage III/IV WT in PT. CD68 and M2 correlate with WT stage and poor survival in both PT and UT.

Table S2 – List of antibodies

Target	Clone	Company	Catalog number	Dilution	# of slides	# of cases per subtypes BLA	EPI	MES	MIX	REG
PD-L1	E1L3N	Cellsignaling	13684	1:200	16	2	1	1	1	3
CD4	EP204	Cellsignaling	48274	1:100	32	5	4	3	9	10
CD8a	C8/144B	E-Biosciences	14-0085	1:400	48	9	4	8	13	11
CD206	685645	R&D systems	MAB25341	1:150	24	4	2	5	4	6
Foxp3	236A/E7	eBiosciences	14-4777-82	1:100	16	2	3	3	3	5
CD86	E2G8P	Cellsignaling	91882S	1:400	16	5	1	3	2	5
IDO1	H-11	Santa Cruz	B0822	1:100	16	3	1	4	2	4
CD68	PG-M1	Dako M	M0876	1:100	48	9	4	8	13	11
CD20	L26	Leica Biosystems	M0755	1:400	24	3	3	3	3	8

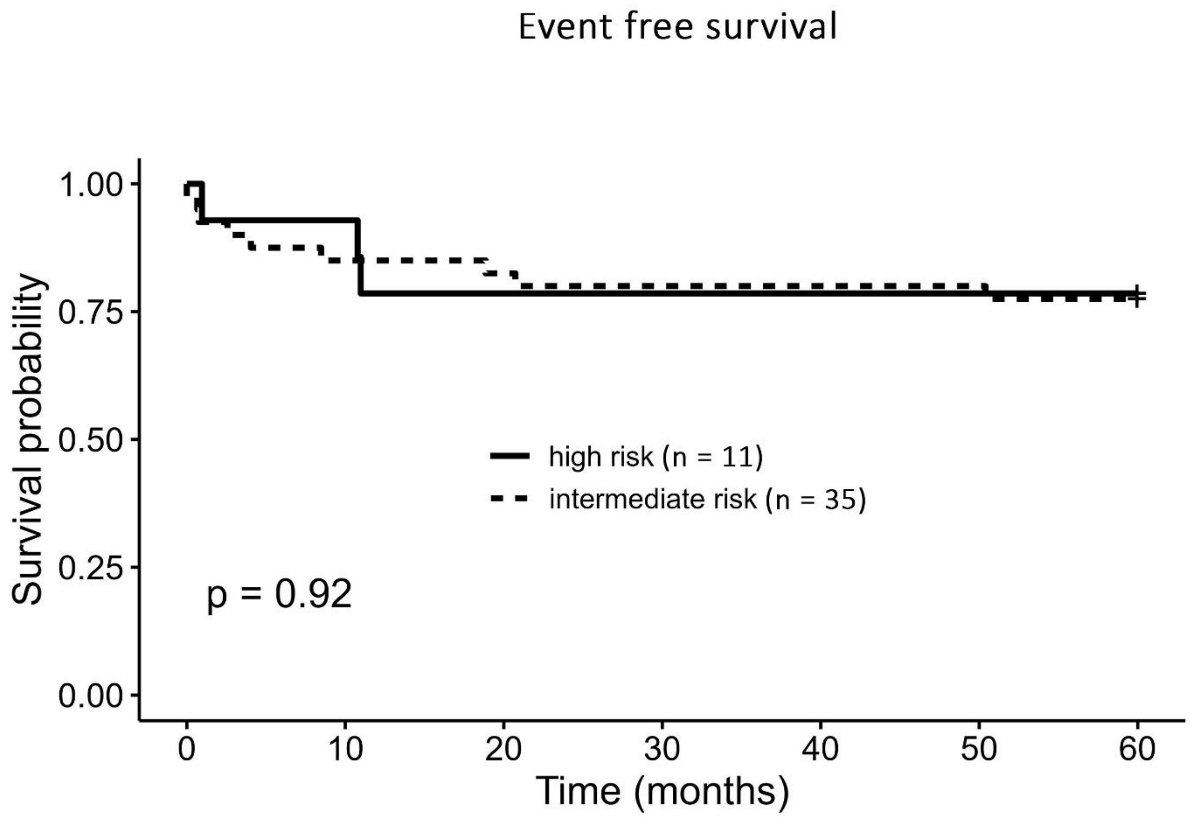

Figure S1 – Kaplan Meier curve plot of intermediate risk patients and high risk patients in our cohort.

Kaplan–Meier curves for event-free survival in intermediate-risk (n = 35) and high-risk (n = 11) patients. No significant difference was detected between groups (log-rank P = 0.92). Given the small cohort and low number of events, this analysis is underpowered and should be interpreted with caution.

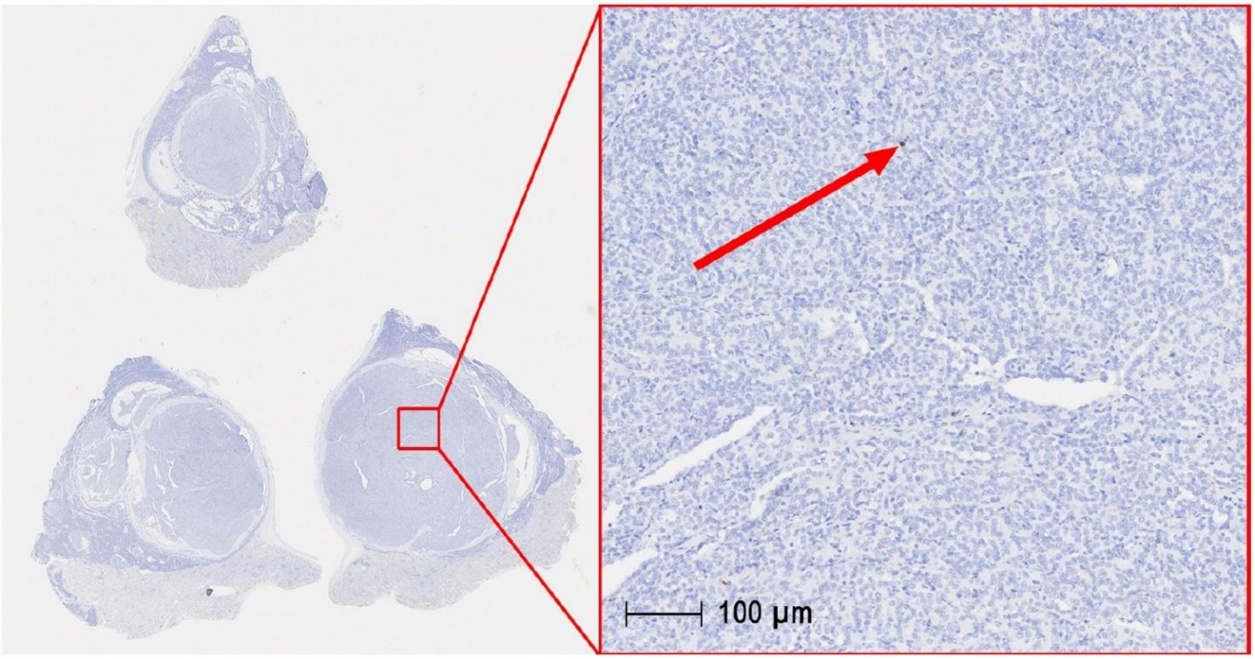

Figure S2 – scarcity of PDL1 positive cells

Image depicting whole slide scans of PDL1-DAB stained section. On the left showing the whole slide with the zoomed our region marked in red. The right side showing the zoomed out region with one single DAB positive cell.

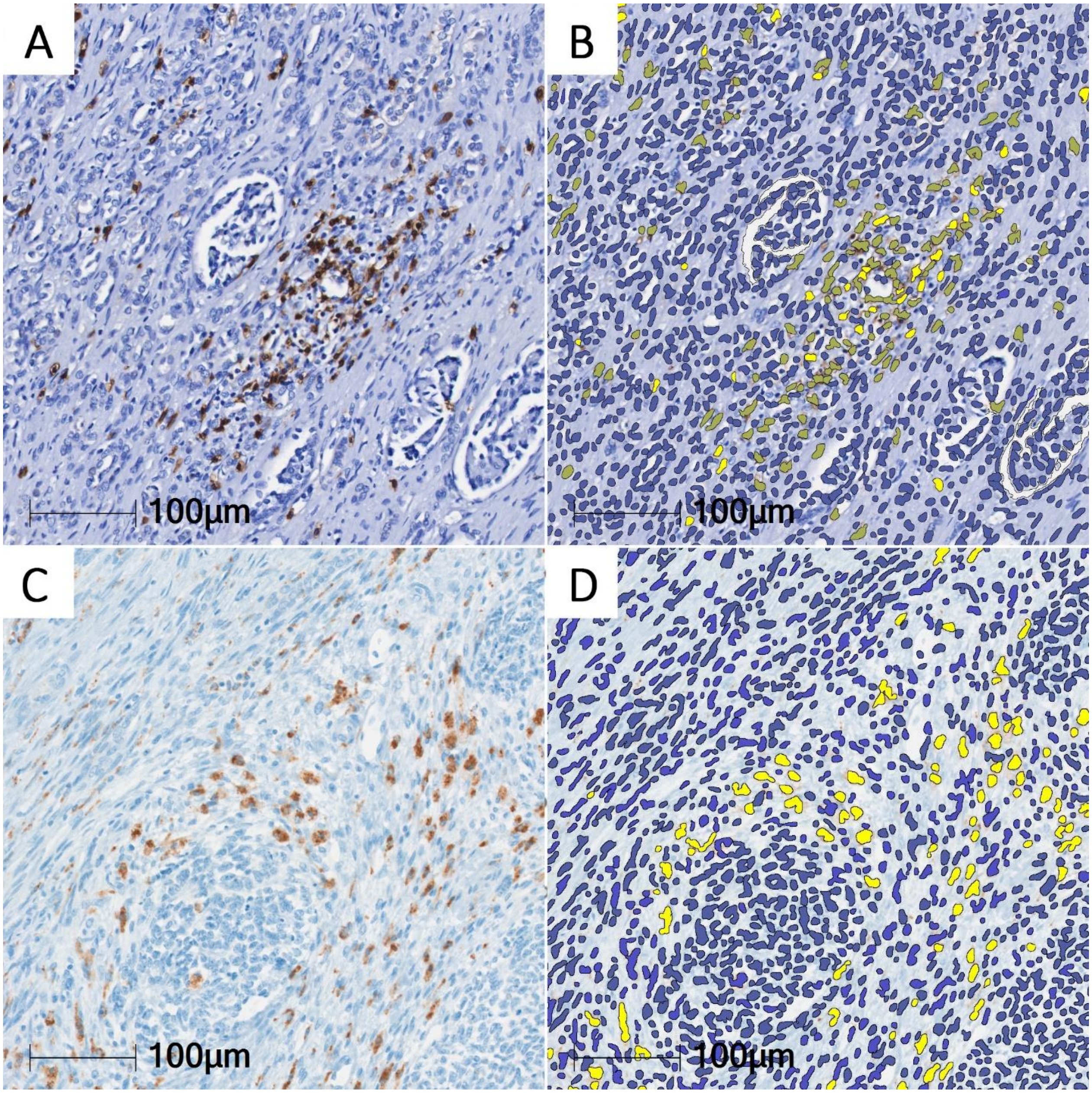

Figure S3 – DensenetV2 tissue classification of blastemal and mesenchymal components

Low magnification view of blastemal and mesenchymal regions of WT with IHC-CD8 (A, B) and IHC-CD68 (C, D) DAB stains, overlayed with DensenetV2 annotations (red: blastema, light-blue: mesenchyme, white: glas).

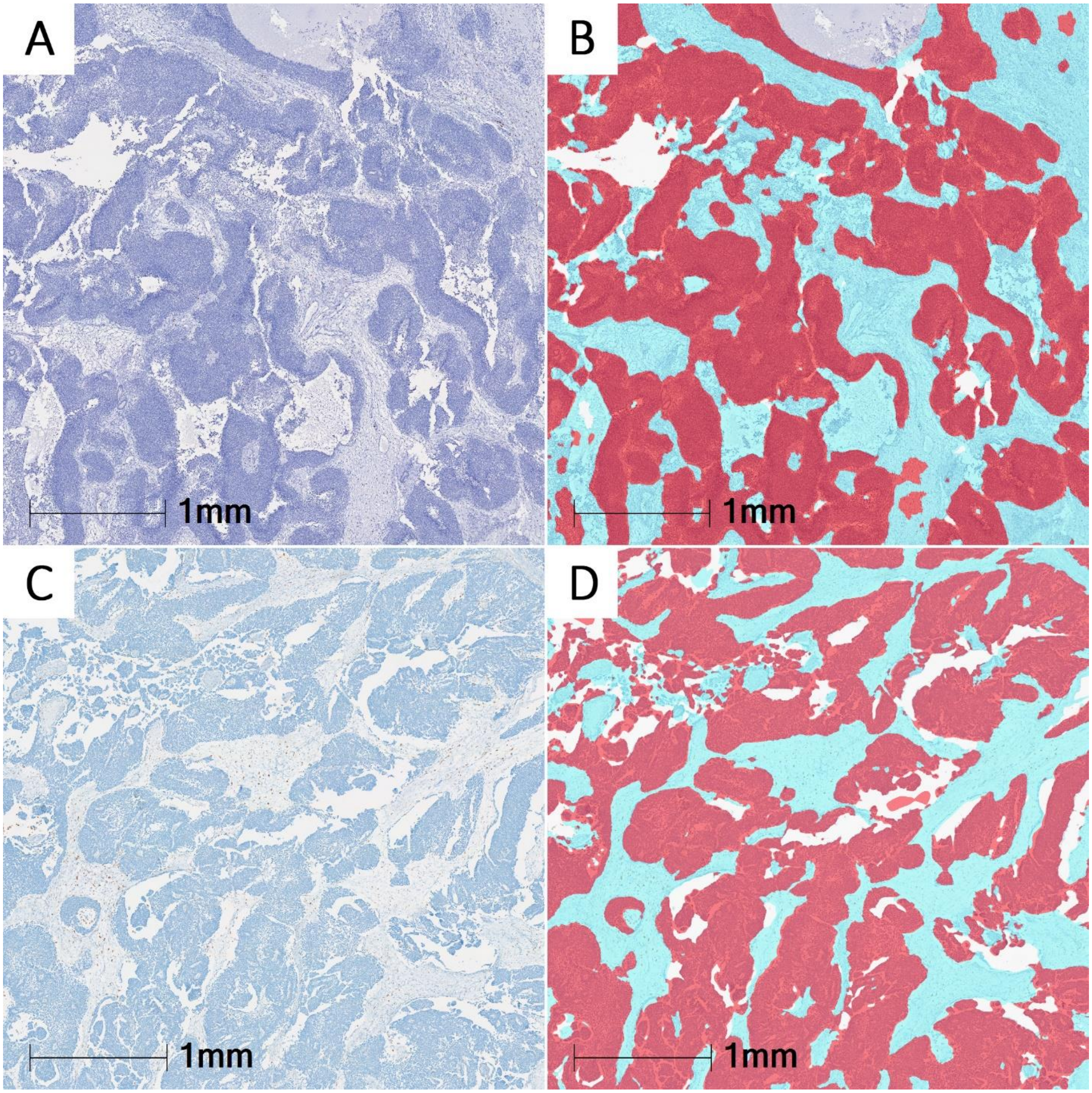

Figure S4 - DensenetV2 tissue classification of epithelial and mesenchyme components

Low magnification view of blastemal and mesenchymal regions of WT with IHC-CD8 (A, B) and IHC-CD68 (C, D) DAB stains, overlayed with DensenetV2 annotations (purple: epithelial components, light-blue: mesenchyme, white: glas

REFERENCES

1. Mardanpour K, Rahbar M, Mardanpour S, Mardanpour N, Rezaei M. CD8+ T-cell lymphocytes infiltration predict clinical outcomes in Wilms' tumor. Tumour Biol. 2020;42(12):1010428320975976.
2. Holl EK, Routh JC, Johnston AW, Frazier V, Rice HE, Tracy ET, Nair SK. Immune expression in children with Wilms tumor: a pilot study. J Pediatr Urol. 2019;15(5):441 e1- e8.
3. Tian K, Du G, Wang X, Wu X, Li L, Liu W, Wu R. MMP-9 secreted by M2-type macrophages promotes Wilms' tumour metastasis through the PI3K/AKT pathway. Mol Biol Rep. 2022;49(5):3469-80.
4. Tian K, Wang X, Wu Y, Wu X, Du G, Liu W, Wu R. Relationship of tumour-associated macrophages with poor prognosis in Wilms' tumour. J Pediatr Urol. 2020;16(3):376 e1- e8.
5. Maturu P, Overwijk WW, Hicks J, Ekmekcioglu S, Grimm EA, Huff V. Characterization of the inflammatory microenvironment and identification of potential therapeutic targets in wilms tumors. Transl Oncol. 2014;7(4):484-92.
6. Liou P, Bader L, Wang A, Yamashiro D, Kandel JJ. Correlation of tumor-associated macrophages and clinicopathological factors in Wilms tumor. Vasc Cell. 2013;5(1):5.
7. Yadav DK, Vishesh J, Dinda AK, Agarwala S. Tumor-Infiltrating Lymphocytes in Wilms Tumor. Indian J Med Paediatr Oncol. 2020;41(1):34-8.
8. Wang Z, Jin L, Wang J, Tian X, Mi T, Li M, Zhang Z, Wu X, Li M, Liu J, et al. Recruitment and polarization typing of tumor-associated macrophages is associated with tumor progression and poor prognosis in Wilms tumor patients. PLoS One. 2024;19(11):e0309910.
